# Differences in oxidative metabolism modulation induced by ischemia/reperfusion between trained and untrained individuals assessed by NIRS

**DOI:** 10.14814/phy2.13384

**Published:** 2017-10-16

**Authors:** Rogério N. Soares, Kaitlin M. McLay, Mitchell A. George, Juan M. Murias

**Affiliations:** ^1^ Faculty of Kinesiology University of Calgary Calgary Alberta Canada

**Keywords:** Oxygen consumption, Blood flow occlusion, Fitness

## Abstract

Endurance training is associated with skeletal muscle adaptations that regulate the oxidative metabolism during ischemia/reperfusion. The aim of this study was to noninvasively assess in vivo differences in the oxidative metabolism activity during ischemia/reperfusion between trained and untrained individuals, using near infrared spectroscopy (NIRS) combined with a vascular occlusion test (VOT) technique (NIRS‐VOT). Sixteen untrained (26.3 ± 5.1 year) and seventeen trained (29.4 ± 4.9 year) healthy young adult men were submitted to a VOT (2 min baseline, 5 min occlusion, and 8 min reperfusion). Oxygen utilization was estimated from the area under the curve of the NIRS‐derived deoxyhemoglobin [HHb] signal during occlusion (AUCocc). Muscle reperfusion was derived from the area above the curve (AACrep) of the [HHb] signal after cuff release. The AUCocc of the untrained participants (21010 ± 9553 % · s) was significantly larger than the AUCocc of their trained counterparts (12320 ± 3283 % · s); *P* = 0.001). The AACrep of the untrained participants (5928 ± 3769 % · s) was significantly larger than the AACrep of the trained participants (3745 ± 1900 % · s; *P* = 0.042). There was a significant correlation between AUCocc and AACrep (*r* = 0.840; *P* = 0.001). NIRS assessment of oxidative metabolism showed that trained individuals are more efficient in shifting between oxidative and anaerobic metabolism in response to ischemia and reperfusion.

## Introduction

In response to hypoxia, anaerobic metabolism becomes upregulated causing a decrease in cellular pH, reduction in cellular ATP, and an increase in oxidative stress (as hypoxia impairs mitochondrial function) (Zhou et al. 1996; Hoppeler et al. 2003). Previous studies have shown that during periods of oxygen starvation (i.e., blood flow occlusion) the skeletal muscle cells upregulate hypoxia‐inducible factors (HIFs) (Stroka et al. 2001; Zagórska and Dulak 2004). Hypoxia‐inducible factors are transcription factors that sense a decrease in oxygen partial pressure and activate genes associated with anaerobic metabolism. This transition away from oxidative metabolism provides a protective effect in response to hypoxia by reducing reactive oxygen species (ROS) production and cell death (Zagórska and Dulak 2004; Lindholm and Rundqvist 2016).

Although fundamental in returning to homeostasis after a period of oxygen deprivation, immediate reperfusion can also have some detrimental effects to the cells (Kalogeris et al. 2012; Peiyuan et al. 2016). The abundance of oxygen during reperfusion, combined with the mitochondrial impairment due to the hypoxia, results in an increased ROS production, which in turns causes more apoptosis (Yellon and Hausenloy 2007). Interestingly, studies have shown that previous exposure to ischemia/reperfusion challenges induce intracellular adaptations that attenuate the damage induced by subsequent arterial occlusions (Wegener et al. 2004; Chan et al. 2005; Walsh et al. 2009). In this sense, the increase in metabolic demand and consequent oxygen consumption by skeletal muscles during exercise performed at intensities above ~70–80% of maximum oxygen uptake reduces the partial pressure in the active muscle (Desplanches et al. 1993; Richardson et al. 1995). This intermittent hypoxia induced by exercise facilitates cellular adaptations associated with a higher tolerance to hypoxia, eliciting a protective role against further ischemia/reperfusion damage (Desplanches et al. 1993; Richardson et al. 1995; Lindholm and Rundqvist 2016). For instance, it has been shown that hypoxia‐induced factor (HIF)‐1 is expressed immediately after an incremental test to exhaustion in skeletal muscle and that it activates the expression of glycolytic enzymes, such as lactate dehydrogenase A and glucose transporters (Mason et al. 2004). Furthermore, the skeletal muscle‐specific deletion of HIF‐1*α* has been associated with higher skeletal muscle damage after performing activities of vigorous intensity (i.e., a reduction in the oxygen tension of the skeletal muscle), reinforcing the idea that exercise protects against the detrimental effects of ischemia (Richardson et al. 1995; Lundby et al. 2006).

Interestingly, HIF seems to be downregulated in skeletal muscle of chronic endurance trained athletes in the normoxic state (Mason et al. 2004). This could result in endurance athletes having a greater reliance on the oxidative metabolism in homeostasis (i.e., resting, and during steady‐state exercise). Additionally, a previous study using near infrared spectroscopy (NIRS) to assess differences in the oxidative metabolism between trained and untrained individuals showed that trained individuals had higher efficiency to activate the oxidative metabolism after twitch electrical stimulations combined with short periods (10–30 sec) of vascular occlusion tests (VOT) (Brizendine et al. 2013). Although this model provided important information in relation to mitochondrial function in trained versus untrained individuals, the use of blood flow occlusions no longer than 30 sec to measure muscular oxygen consumption might not be sufficient to significantly decrease the partial pressure of oxygen and consequently induce metabolic adaptations in the skeletal muscle. For example, previous studies measuring oxygen saturation during VOTs have shown that desaturation does not reach a plateau until close to 5 min of occlusion, at which point a greater expression of HIF may be detectable between groups. (McLay et al. 2016b; Soares et al. 2017).

Therefore, the aim of this study was to noninvasively assess in vivo whether trained individuals were more efficient at acutely modulate the oxidative metabolism activity of the skeletal muscle during ischemia/reperfusion compared with untrained individuals, using NIRS combined with a VOT (NIRS‐VOT).

## Methods

### Ethical Approval

This study was approved by the Conjoint Health Research Ethics Board of the University of Calgary. The study adhered to the principles established in the declaration of Helsinki.

### Participants

Sixteen untrained (26 ± 5 year) and seventeen trained (29 ± 4 year) healthy young adult men volunteered and gave written informed consent to participate in the study. All participants were free of disease and none of them were taking any medications that would alter hemodynamic responses to the occlusion/reperfusion test. Additionally, all participants were nonsmokers. Participants were classified into two groups, trained and untrained, based on self‐reported cardiovascular training and results of a ramp incremental (RI) exercise test. All trained individuals reported regular cardiovascular training over a number of years and their cardiovascular fitness attested to their reports. It should be noted that all trained individuals had a maximal oxygen uptake (*V̇*O_2max_) greater than 55 mL/(min kg) (Pauw et al. 2013). Conversely, untrained individuals reported participation in some physical activities but did not participate in any regular training programs and their *V̇*O_2max_ was average.

### Experimental procedure

Participants reported to the laboratory on two separate occasions. Participants were asked to fast for 2 h and refrain from caffeine and alcohol for 12 h before each visit. On the first visit, participants performed a ramp incremental exercise test to exhaustion (20 W baseline for 4 min followed by a 25 W/min ramp) on a magnetically braked cycle ergometer (Velotron RacerMate Inc., Seattle, WA) for determination of *V̇*O_2max_. The *V̇*O_2max_ was defined as the greatest 20 sec *V̇*O_2_ computed from a rolling average during the last minute of the ramp incremental test, and peak power output was defined as the power output achieved at termination of the ramp incremental test.

Participants returned to the laboratory on a separate day to complete a vascular occlusion test. As previously described (McLay et al. 2016a), participants were asked to lay supine on an examination table for 10 min. Following the 10‐min rest‐period, the NIRS probe was placed on the muscle belly of the tibialis anterior, secured via a tightened black elastic strap to mitigate movement, and covered with an optically dense black vinyl sheet to minimize the intrusion of extraneous light. An elastic tensor bandage was then loosely wrapped around the site, as not to constrict blood flow, but to further minimize movement and light intrusion. The probe remained in place for the duration of testing. A pneumatic cuff connected to an automatic rapid inflation system (Hokanson E20 AG101, Bellevue, WA) was placed below the knee (approximately 5 cm distal to the popliteal fossa) and used to induce the occlusion of blood flow. The cuff was inflated to 250 mmHg for the occlusion period. NIRS measurements were collected continuously at an output frequency of 2 Hz for the duration of the VOT (2 min of baseline, 5 min of occlusion, and 8 min following cuff release) (McLay et al. 2016a).

### Near‐infrared spectroscopy

Deoxygenated hemoglobin concentration ([HHb]) of the tibialis anterior muscle was monitored continuously throughout each VOT with a frequency‐domain multidistance NIRS system (Oxiplex TS, ISS, Champaign, Ill.,). Briefly, the system was composed of a single channel consisting of 8 laser diodes operating at 2 wavelengths (*λ *= 690 and 828 nm, 4 at each wavelength), which were pulsed in rapid succession. The lightweight plastic NIRS probe (connected to laser diodes and a photomultiplier tube by optical fibers) consisted of 2 parallel rows of light emitter fibers and 1 detector fiber bundle; the source‐detector separations for this probe were 2.0, 2.5, 3.0, and 3.5 cm for both wavelengths. By measuring changes in light absorption at different wavelengths, changes in [HHb] can be measured continuously. The NIRS device was calibrated at the beginning of the session following an instrument warm‐up period of at least 30 min. The calibration was performed with the probe placed on a calibration block (phantom) with absorption (*μ*_a_) and reduced scattering coefficients (*μ*_s’_) previously measured. Thus, correction factors were determined and were automatically implemented by the manufacturer's software for the calculation of the *μ*_a_ and *μ*_s’_ for each wavelength during the data collection. Calculation of [HHb] reflected continuous measurements of *μ*_s’_ made throughout each testing session (i.e., constant scattering value not assumed). The data were sampled at 2 Hz and then further averaged to 1 sec bins.

**Figure 1:**
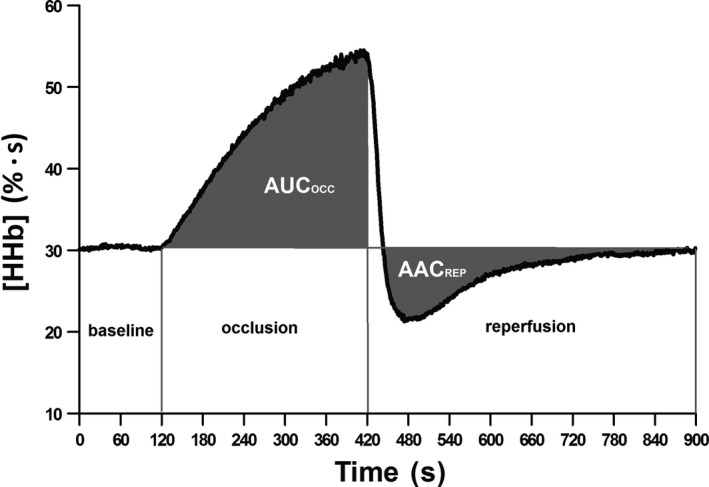
Representative profile of the deoxyhemoglobin ([HHb]) signal analysis during and subsequent to cuff occlussion.Baseline, 2 min period previous to the occlusion; occlusion, five min period during cuff inflation at 250 mmHg; reperfusion, 8 min period after cuff release; AUCocc, area under the curve of deoxyhemoglobin during occlusion period; AACrep, area above the curve of deoxyhemoglobin during reperfusion.

The [HHb] signal has been previously used to measure oxidative metabolism (Ryan et al. 2014). The baseline of the [HHb] signal was calculated as the average of the last 2 min prior to blood flow occlusion. The [HHb] area under the curve during occlusion (AUCocc) was calculated as the total area under the curve of percent variation in the [HHb] signal, from the baseline value to the end of the 5 min before cuff release (Fig. 1). Using an occlusion model and in the absence of blood flow, an increase in the [HHb] signal represents an increase in O_2_ consumption in the area of NIRS interrogation. The [HHb] area above the curve (AACrep) was calculated as the total area above the curve of percent variation in the [HHb] signal from the cuff inflation until the end of the 8 min post cuff release (Fig. 1). During the postocclusion period, the adjustment of the [HHb] signal reflects the balance between oxygen delivery and oxygen utilization and provides information about the rate of reperfusion. For both AUCocc and AACrep calculations, the baseline value for each test represented 0% of variation.

### Statistical analysis

Data are presented as mean ± standard deviation (SD). All data were tested for normality using the D'Agostino & Pearson normality test. An unpaired Student‐*T* test was applied to compare the anthropometric characteristics and main variables between groups. Pearson Product Moment Correlation test was performed to analyze the correlation between area under the curve of deoxyhemoglobin during occlusion and area above the curve of deoxyhemoglobin during reperfusion. A *P* < 0.05 was considered as the level of statistical significance. Data analysis was performed using Graphpad Prisma 7.0.

## Results

**Figure 2:**
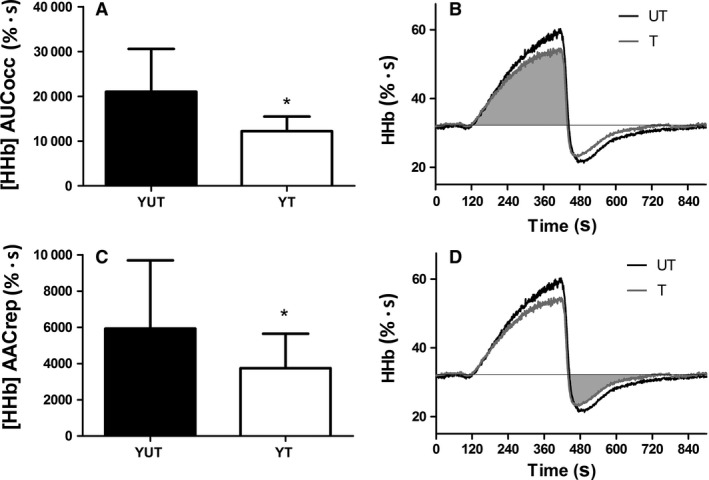
Difference between young untrained and trained individuals for the area under the curve of deoxyhemoglobin signal during occlusion (AUCocc) and area above the curve of deoxyhemoglobin signal during reperfusion (AACrep). Panel A shows the smaller area under the curve of deoxyhemoglobin signal during occlusion period of trained compared to the untrained participants (mean ± SD). Panel B depicts the average profile of the deoxyhemoglobin signal during the vascular occlusion test of trained and untrained participants, highlighting the smaller area under the curve of deoxyhemoglobin during occlusion period in the trained participants (filled in gray) when compared to untrained individuals. Panel C shows the smaller area above the curve of deoxyhemoglobin during reperfusion period of trained participants when compared to their untrained counterparts (mean ± SD). Panel D depicts the smaller area above the curve of deoxyhemoglobin during reperfusion of trained compared to untrained participants (filled in gray). *Different from young untrained (*P* < 0.05)

The participants’ characteristics according to groups are described in table 1. The age and BMI of the participants was not significantly different (*P* > 0.05). Trained individual achieved a greater *V*O_2max_ and peak power output compared to their untrained counterparts (*P* < 0.05). The area under the curve of deoxyhemoglobin during occlusion of the untrained participants (21010 ± 9553 % · s) was significantly larger than the area under the curve of deoxyhemoglobin during occlusion of their trained counterparts (12320 ± 3283 % · s; *P* = 0.001) (Figure 2, Panels A and B).

**Table 1:** Participant characteristics (Mean ± SD)

Variables	Untrained *n* = 16	Trained *n* = 17
Age (years)	26.3 ± 5.10	29.4 ± 4.90
BMI (kg/m^2^)	25.4 ± 4.0	23.8 ± 2.1
Relative *V*O_2max_ (mL/kg per min)	39.0 ± 4.6	59.8 ± 3.5^a^
Peak power output	272 ± 48	410 ± 38^a^

BMI, body mass index.

a Difference between groups (*P* < 0.05).

**Figure 3:**
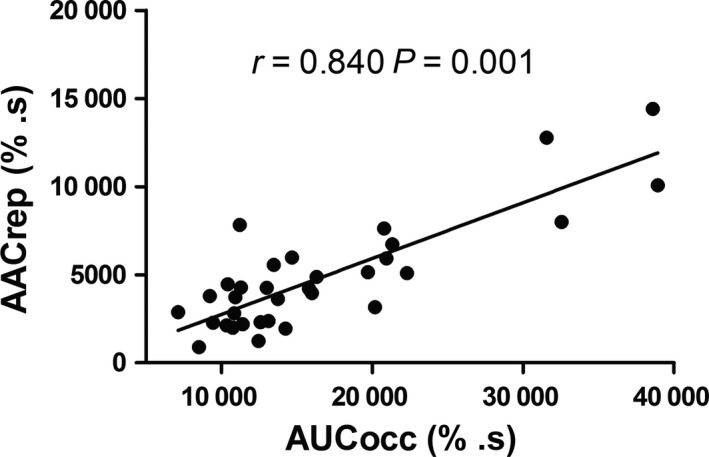
Correlation between the area under the curve of deoxyhemoglobin signal during occlusion period (AUCocc) and the area above the curve of deoxyhemoglobin signal during the reperfusion period (AACrep).

The area above the curve of deoxyhemoglobin during reperfusion of the untrained participants (5928 ± 3769 % · s) was significantly larger than the area above the curve of deoxyhemoglobin during reperfusion of the trained participants (3745 ± 1900 % · s; *P* = 0.042) (Fig. 2, Panels C and D). There was a significant correlation between area under the curve of deoxyhemoglobin during occlusion and area above the curve of deoxyhemoglobin during reperfusion (*r* = 0.840; *P* = 0.001).

## Discussion

The capacity to shift from aerobic to anaerobic metabolism in situations where oxygen provision is impaired, and the ability to rapidly activate the oxidative metabolism after a period of oxygen starvation are important for tissue survival (Zagórska and Dulak 2004). The findings from the present study indicate that trained individuals are more efficient in shifting from oxidative to anaerobic metabolism during an ischemic period compared with their untrained counterparts. Additionally, the lower reliance on deoxyhemoglobin after cuff release in the trained individuals suggested a more rapid and efficient reperfusion in these participants, which might contribute to a faster shift back to aerobic metabolism compared to untrained individuals.

This study showed a smaller area under the curve of deoxyhemoglobin during occlusion during hypoxia in the trained group, suggesting a reduced reliance on oxidative metabolism. This indicates an increased efficiency in shifting from oxidative to anaerobic metabolism in trained compared with untrained individuals, which can be evaluated using a noninvasive technique. Although thisstudy did not examine the mechanisms responsible for this response, these data are in agreement with a previous study examining chronic cellular adaptations to exercise that suggested daily drops in intramyocellular oxygen tension at the onset of exercise might reflect an increased potential for transient activation of the hypoxia‐inducible factors‐1 during exposure to hypoxia and upregulation of anaerobic enzymes of the glycolytic pathway (Hoppeler et al. 2003). During ischemia, cellular mechanisms become dysfunctional, the mitochondrial calcium levels increase (calcium overload) causing cell swelling, rupture, and apoptosis (Kalogeris et al. 2012). In this sense, Ong et al. (2014) showed that the higher hypoxia‐inducible factors‐1 activation observed in athletes, was associated with protection against cellular damage during ischemia/reperfusion by promoting aerobic glycolysis and decreasing mitochondrial oxidative stress production and apoptosis (Ong et al. 2014).

Among other cellular adaptations (Fernandes et al. 2012; Green et al. 2014) the “hypoxic environment” induced by the onset of vigorous exercise activates hypoxia‐inducible factors (Stroka et al. 2001). As mentioned earlier, HIFs are hypoxia‐sensitive transcription factors that upregulate genes involved in the expression of anaerobic metabolism enzymes (such as, lactate dehydrogenase, glucose transporter 1 and 4, hexokinase 1 and 2), consequently reducing the oxidative metabolism activity (Zagórska and Dulak 2004; Lindholm and Rundqvist 2016). Previous studies showed that the hypoxia‐inducible factors are heterodimers composed by a nuclear subunit and three O_2_‐regulated HIF‐*α* cytosolic subunits, named HIF‐1*α*, HIF‐2*α* and HIF‐3*α* (Schofield and Ratcliffe 2004; Thomas et al. 2016). Their expression is mainly regulated by oxygen‐sensitive factors named prolyl hydroxylases. In normal oxygen conditions, prolyl hydroxylases are active and induce degradation of hypoxia‐inducible factors, thereby inhibiting anaerobic metabolism and activating oxidative metabolism (Zagórska and Dulak 2004; Thomas et al. 2016). However, the decrease in the oxygen partial pressure during hypoxia inactivates prolyl hydroxylases, leading to the stabilization of hypoxia‐inducible factors and triggering the expression of specific target genes related to anaerobic enzymes (Zagórska and Dulak 2004; Thomas et al. 2016).

Moreover, the present study also showed that trained individuals showed a more efficient reperfusion (smaller area above the curve of deoxyhemoglobin during reperfusion) than untrained individuals, which may improve the ability to switch back from anaerobic to aerobic metabolism. Exercise training is associated with improvements in vascular parameters (i.e., vascular responsiveness, endothelial function) that are associated to better reperfusion after a blood flow occlusion period (Delp 1995; Clarkson et al. 1999; McLay et al. 2016b). For instance, McLay et al. 2016b showed that the reperfusion slope of oxygen saturation was steeper (better vascular responsiveness) in trained when compared with untrained individuals after 30 sec, 1, 2, 3, and 5 min of blood flow occlusion. Additionally, in agreement with the present findings, a previous study using biopsies of the vastus lateralis found that elite athletes showed significantly higher expression of prolyl hydroxylases than the moderately active individuals (Lindholm and Rundqvist 2016), which suggests an improved ability to switch from anaerobic to aerobic metabolism. The better efficiency to provide oxygen to the cells after an ischemic period and the overexpression of enzymes associated with higher oxidative metabolism activity might result in a faster oxidative metabolism activation after cuff release observed in our study. In this sense, Murias et al. (2010) showed that 3 weeks of endurance training was sufficient to “speed up” the rate of adjustment of the oxidative metabolism to a new metabolic demand during the onset of exercise (Murias et al. 2010), reinforcing the association between training status and efficiency in activating oxidative metabolism in response to a new metabolic state and the protective role of exercise. The correlation between AACrep and AUCocc found in this study showed that the individuals that had lower oxidative metabolism activation during occlusion, likely had a greater oxidative metabolism activity as reflect by greater reliance on oxygen extraction during reperfusion period. This finding also reinforce the idea of the effects of training in the sensitivity to changes in oxygen concentration. Furthermore studies examining the mechanisms underlying the NIRS‐derived assessment of oxidative metabolism are warranted to better identify these protective microvascular adaptations induced by endurance training in the skeletal muscle of trained individuals.

### Limitations of the study

The amplitude of NIRS signal has been shown to be affected by the adipose tissues thickness (ATT) underneath the site of placement of the probe (Ohmae et al. 2014). However, it has been indicated that the profile of the [HHb] signal remains unaffected by ATT as long as this value is not greater than the penetration depth of the NIIR light (Grassi and Quaresima 2016). Although not measured in the present study, data from other experiments in our laboratory indicate that the ATT underneath the tibialis anterior is rarely in excess of 10 mm in young nonobese individuals. This value is far below the penetration depth of the NIR light into the tissue. Additionally, given the normalization strategy used in this study, the absolute amplitude of the signal becomes arbitrary. Additionally, changes in the [HHb] reflect deoxygenation of both hemoglobin and myoglobin. Although the precise contribution of each one of these components to the total [HHb] signal is unknown, it has been indicated that the total contribution of myoglobin to the [HHb] signal is ~10% (Grassi and Quaresima 2016), and it is thus assumed that the large majority of the changes in the [HHb] signal are attributable to changes at the concentration of deoxygenated hemoglobin. Finally, training‐related differences in muscle fiber composition that were not evaluated in this study might affect the responses. However, (1) it is important to note that the [HHb] baseline measurements were not different between trained and untrained (same oxidative metabolism activity before the occlusion), suggesting that, at least at rest, a potential difference in muscle fiber type composition between groups was not affecting the responses; (2) the tibialis anterior of the trained group showed lower oxidative metabolism activity during blood flow occlusion, but also showed higher activity during reperfusion. This cannot be explained by differences in fiber types between groups, but by differences in the sensitivity to changes in oxygen availability.

In conclusion, this study demonstrated that the noninvasive NIRS‐derived assessment of oxidative metabolism in the skeletal muscle throughout a VOT showed that trained individuals are more efficient in shifting between oxidative and anaerobic metabolism in response to ischemia and reperfusion.

## Conflict of Interest

The authors declare that there is no conflict of interest regarding to competing interests, author contributions and Funding.
